# RKIP regulates CCL5 expression to inhibit breast cancer invasion and metastasis by controlling macrophage infiltration

**DOI:** 10.18632/oncotarget.5176

**Published:** 2015-08-13

**Authors:** Ila Datar, Xiaoliang Qiu, Hong Zhi Ma, Miranda Yeung, Shweta Aras, Ivana de la Serna, Fahd Al-Mulla, Jean Paul Thiery, Robert Trumbly, Xuan Fan, Hongjuan Cui, Kam C. Yeung

**Affiliations:** ^1^ Department of Biochemistry and Cancer Biology, University of Toledo, College of Medicine, Health Science Campus, Toledo, OH, USA; ^2^ Kuwait University, Faculty of Medicine. P.O. Box 24923, Safat, Kuwait; ^3^ Department of Biochemistry, Yong Loo Lin School of Medicine, National University of Singapore, Singapore; ^4^ State Key Laboratory Of Silkworm Genome Biology, Chongqing, China

**Keywords:** metastasis suppressor, RKIP, tumor microenvironment, CCL5

## Abstract

Accumulating evidence suggests that presence of macrophages in the tumor microenvironment add to the invasive and tumor-promoting hallmarks of cancer cells by secreting angiogenic and growth factors. RKIP is a known metastasis suppressor and interferes with several steps of metastasis. However, the mechanistic underpinnings of its function as a broad metastasis suppressor remain poorly understood. Here, we establish a novel pathway for RKIP regulation of metastasis inhibition through the negative regulation of RANTES/CCL5 thereby limiting tumor macrophage infiltration and inhibition of angiogenesis. Using a combination of loss- and gain-of-function approaches, we show that RKIP hinders breast cancer cell invasion by inhibiting expression of the CC chemokine CCL5 *in vitro*. We also show that the expression levels of RKIP and CCL5 are inversely correlated among clinical human breast cancer samples. Using a mouse allograft breast cancer transplantation model, we highlight that ectopic expression of RKIP significantly decreases tumor vasculature, macrophage infiltration and lung metastases. Mechanistically, we demonstrate that the inhibition of the CCL5 expression is the cause of the observed effects resulting from RKIP expression. Taken together, our results underscore the significance of RKIP as important negative regulator of tumor microenvironment.

## INTRODUCTION

Tumors are complex, heterotypic masses, which comprise in addition to cancer cells, the cells of the neoplastic stroma. The cellular milieu in which these stroma cells interact with cancer cells, with one another and with the extracellular matrix is known as the tumor microenvironment. The plasticity of the tumor microenvironment may provide conducive factors, which add to the invasive and metastatic spread of cancer cells [1-3]. Among the many cells found in the neoplastic stroma, macrophages play a pivotal role in shaping the tumor microenvironment and providing growth and survival signals for the evolving cancer cells [4, 5]. The macrophages that associate with tumors have been shown both experimentally and clinically to enhance tumor progression to malignancy [2, 6]. Tumor cells express several inflammatory chemokines, cytokines and growth factors to recruit macrophages and support their growth [7]. Among them, the CC chemokines RANTE/CCL5 is as one of the key chemotactic factors for macrophages in breast cancer. It was shown that tumor cell-derived CCL5 promoted breast cancer by recruiting macrophages into the tumor microenvironment.

Once recruited into the tumors, macrophages in turn discharge angiogenetic growth factors and proteases to promote angiogenesis, tumor progression and metastasis. Importantly, the elevated expression levels of CCL5 are directly correlated with a more advanced breast cancer progression in clinic [8-10]. RKIP is a known tumor and metastasis suppressor. The expression of RKIP is decreased in cancers and further reduced in distant metastases [11-18]. Loss of RKIP expression has been an important indicator of poor prognosis in several types of malignancies including breast and prostate cancer [19-21]. Significantly, restoration of RKIP expression inhibits prostate and breast cancer metastasis [13, 22-24] in cancer cells transplantation mouse models. Recently, RKIP expression was also demonstrated to have a measurable impact on cancer initiation and progression in an autochthonous model of prostate cancer [25]. However, the molecular mechanism of how RKIP inhibits cancer initiation and progression is not well understood. Here, we show that RKIP has a causal role in the regulation of CCL5 expression in breast cancer cells. We show that RKIP regulates *in vitro* breast cancer cell invasion by modulating the expression level of CCL5. Using the 4T1 orthotopic transplantation mouse model [23], we observed that expression of RKIP inhibits angiogenesis and distant metastasis while expression of RKIP/CCL5 in combination rescues this effect. We found that ectopic expression of RKIP suppresses recruitment of macrophages to the primary tumors as well as the lung metastases. Importantly, expression of CCL5 reverses the inhibition of macrophages recruitment due to the expression of RKIP. Our current study therefore provides evidence to support a hitherto unknown regulatory role of RKIP in the tumor microenvironment. RKIP expression favors the existence of a more subdued microenvironment for cancer cells by suppressing the expression of chemokine CCL5 and subsequent inhibition of active macrophages recruitment into the tumors.

## RESULTS

### RKIP negatively regulates CCL5 expression in breast cancer cells

**Figure 1:**
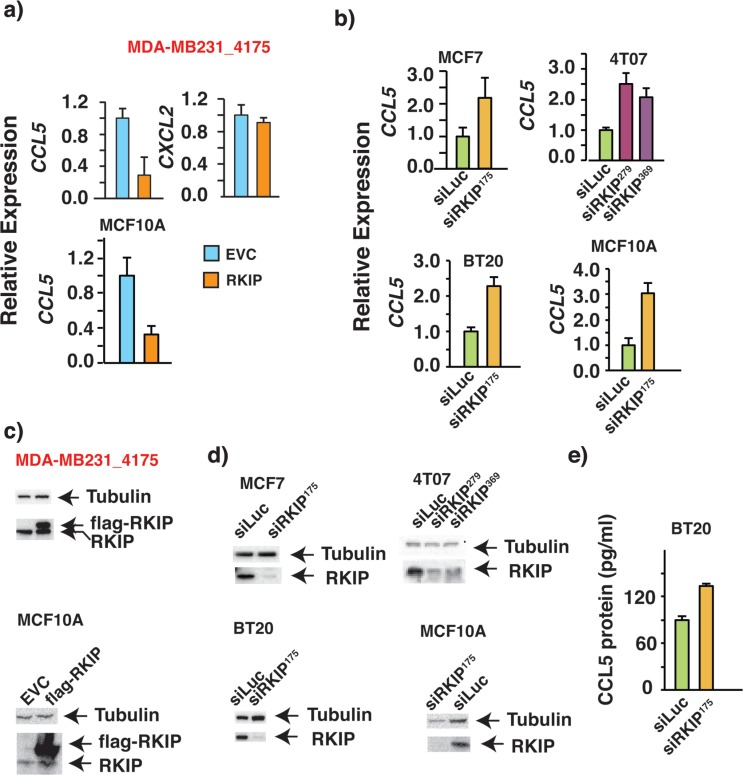
RKIP negatively regulates the expression of RANTES/CCL5 in multiple breast cancer cell lines (a-b) Quantitative real time PCR (qRT-PCR) shows changes in mRNA expression of CCL5 in the indicated cell lines due to the alteration in RKIP expression. The experiment was repeated 3X with similar results (c-d) Western Blots demonstrating the ectopic expression of flag-tagged RKIP and RKIP knock-down by specific siRNAs in indicated breast cell lines with two different siRNAs. (e) ELISA of CCL5 protein expression shows increase in CCL5 protein expression upon stable knockdown of RKIP in BT20 cells.

We previously reported that RKIP was able to modulate the expression of a group of inflammatory chemokines through the NFκB pathway [37]. Of the many chemokines, we chose to investigate the role of RKIP in the regulation of CCL5 expression owing to its involvement in breast cancer development and progression. More importantly CCL5 is a key player in mediating cross-talks between tumor cells and stroma and shifting the balance to a tumor-promoting environment by recruitment of tumor associated macrophages or TAMs [9, 10]. We observed that upon ectopic expression of RKIP in MDA-MB231_4175 cells, the expression of CCL5 mRNA was greatly repressed (Fig. 1a, upper right panel). The effect is specific as the expression of CXCL2 was unaffected by RKIP expression (Fig. 1a, upper right panel). The effect is not cell type specific either since expression of RKIP had a similar effect on CCL5 expression in non-tumorigenic mammary epithelial cell line MCF10A (Fig. 1a, lower panel). We confirmed the expression of RKIP in MDA-MB231_4175 and MCF10A cells by Western blotting and qRT-PCR (Fig.1c and data not shown). Conversely, the expression of CCL5 was increased when RKIP expression was stably knocked down by specific siRNAs in human (MCF7 and BT20) or mouse (4T07) breast cell lines (Fig. 1b, e). Efficiency of RKIP knock-down was determined at protein and mRNA levels (Fig 1d and data not shown). Two different siRNAs were used to knock down RKIP expression to ensure specificity of the knock-down effect on CCL5 expression (Fig. 1b, upper right panel). Our results therefore indicate that RKIP may play a causal role in regulating the expression of CCL5.

### RKIP and CCL5 expression correlate inversely in breast cancer

**Figure 2:**
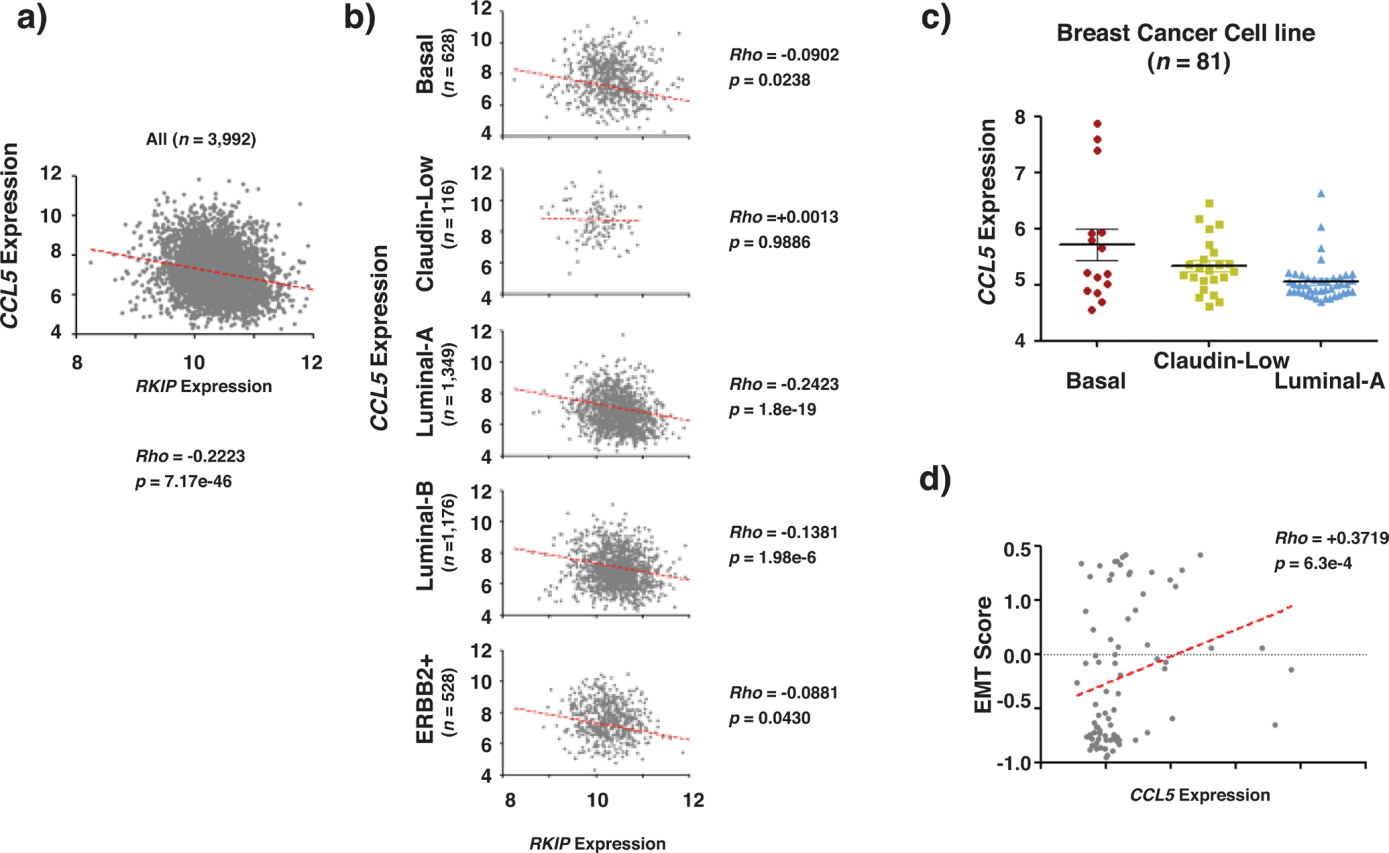
RKIP and CCL5 expression correlate inversely in breast cancer (a-b) Show the inverse correlation between RKIP and *CCL5* expression in clinical breast cancer samples (a) and in clinical breast cancer samples after appropriate molecular sub-classification (b). (c) CCL5 expression in different breast cancer cell lines grouped according to their molecular subtypes. (d) Shows the positive correlation between RKIP expression and EMT scores obtained in breast cancer cell lines. The *p* values compare each subclass with the rest of subclasses. Rho statistic was used to establish the linear correlation between samples.

To explore the relationship between RKIP and CCL5 *in vivo*, mRNA expression of the two genes was examined in 3992 human breast cancers. Their expression was inversely correlated, with p-value < 0.05 (Fig. 2a) in all breast cancer subtypes except for the Claudin-Low (Figure 2b). The expression correlation between RKIP and CCL5 cannot be determined in the Claudin-Low subtype because of the small sample size (n= 116). Previously we reported the expression of RKIP mRNA in 81 samples from 70 breast cancer cell lines [19]. We observed that Luminal breast cancer subtype had the highest RKIP expression levels followed by Basal subtype while Claudin-low subtype had the lowest levels of RKIP expression. Furthermore in agreement with the tumor suppressive role of RKIP we also observed in the same 81 cell lines a significant inverse relationship between RKIP expression and the EMT (epithelial to mesenchymal transition) score, a measure of the EMT phenotype that is frequently associated with aggressive Basal subtype of breast cancer [19]. Consistent with the inverse expression correlation between RKIP and CCL5 in clinical samples, CCL5 expression showed an expression pattern opposite to that reported for RKIP in the same 81 samples from 70 breast cancer cell lines with elevated expression level in Basal, followed by Claudin-Low subtypes and the lowest expression level in the least aggressive Luminal subtype of breast cancer (Fig. 2c). Similarly, we observed a significant and positive correlation between CCL5 expression and the EMT score (Fig. 2d).

### RKIP inhibits breast cancer cell invasion by decreasing CCL5 expression

**Figure 3:**
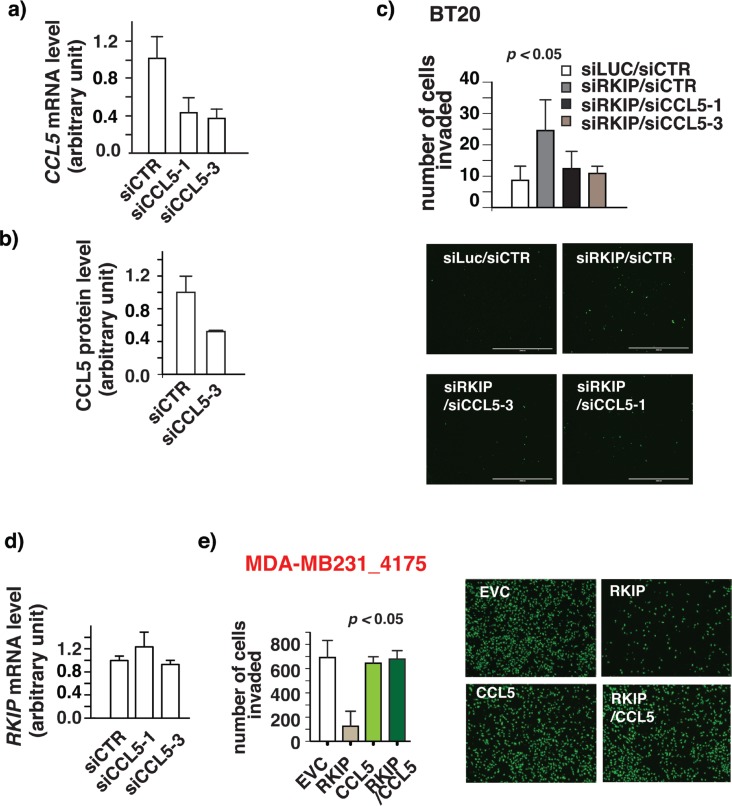
RKIP inhibits breast cancer cell invasion by decreasing CCL5 expression (a) qRT-PCR shows downregulation of *CCL5* expression in cell lines upon stably CCL5 knockdown with two different siRNAs (b) ELISA of CCL5 protein expression shows decrease in CCL5 protein expression upon stable knockdown of CCL5. (c) Increase in invasion by RKIP knockdown is reversed upon knockdown of RKIP and CCL5 together. Breast cancer cells BT20 were infected with siRKIP- or siCCL5-expressing retro viruses or both. siRNA against luciferase (siLuc) or scrambled siRNA (siCTR) was used as negative control. The number of invaded cells was counted 24 hours later using a matrigel-based invasion assay. One way analysis of variance was performed with a P-value=0.0009. It was followed by Bonferroni's multiple comparison test where EVC vs siRKIP, siRKIP vs siRKIP/siCCL5-3 and siRKIP/siCCL5-1 were found to be significant with a p-value<0.05. Right panel, a representative field of matrigel membrane from the right panel with the invaded cells stained in green. (d) Knockdown of CCL5 expression has no effect on *RKIP* expression. Quantitative real time PCR (qRT-PCR) shows changes in mRNA expression of *RKIP* in BT20 cells without or with the knockdown of CCL5 expression. (e) Decrease in invasion upon ectopic expression of RKIP in highly invasive MDA-MB231_4175 cells is reversed upon CCL5 expression. Cells were infected with RKIP- or CCL5-expressing retroviruses or both. The number of invaded cells was counted 24 hours later using a matrigel-based invasion assay. These results are an average of three independent experiments performed in triplicate. Right panel, a representative field of matrigel membrane from the right panel with the invaded cells stained in green. One way analysis of variance was performed with a P-value=0.0035. It was followed by Bonferroni's multiple comparison test where EVC vs RKIP and RKIP vs RKIP/CCL5 were found to be significant with a p-value<0.05. These experiments were performed at least twice in triplicates each time.

The secreted CCL5 can act in an autocrine, paracrine or juxtacrine manner to advance processes crucial for cancer development. It was documented that the autocrine activities of CCL5 promoted breast cancer cells invasion *in vitro* [8, 38]. Knocking down RKIP expression in high-RKIP-expressing breast cancer cells boosts CCL5 expression and increases cell invasion (Fig. 1b and supplementary Fig.) It is possible that the increased CCL5 expression is the cause of the observed increased cell invasion associated with RKIP knockdown in BT20 cells. We reasoned that knocking down CCL5 expression will reverse the effect due to the knockdown of RKIP if increased CCL5 expression is the cause. Indeed, we observed a decrease in invasion in double knockdown cells when compared with control cells (Fig. 3c.) whereas knocking down of CCL5 alone did not affect the invasive capacity of cells (supplementary figure 1). To ensure specificity, we used two different siRNAs to knock-down CCL5 expression (Fig. 3a). We also determined CCL5 expression in the knockdown cells at protein level by ELISA assay (Fig. 3b). Restoration of RKIP expression in low-RKIP-expressing MDA-MB231 cells suppressed cell invasion and inhibited CCL5 expression (Fig 1a). In further support of the notion that CCL5 plays a causal role in RKIP-mediated suppression of breast cancer cell invasion, expression of CCL5 is sufficient to rescue the inhibition of invasion in RKIP expressing 4175 cells while the expression of CCL5 alone had no effect on the invasive capacity of the 4175 cells (Fig. 3d).

### RKIP inhibits angiogenesis and infiltration of macrophages in primary tumors by inhibiting CCL5 expression

**Figure 4:**
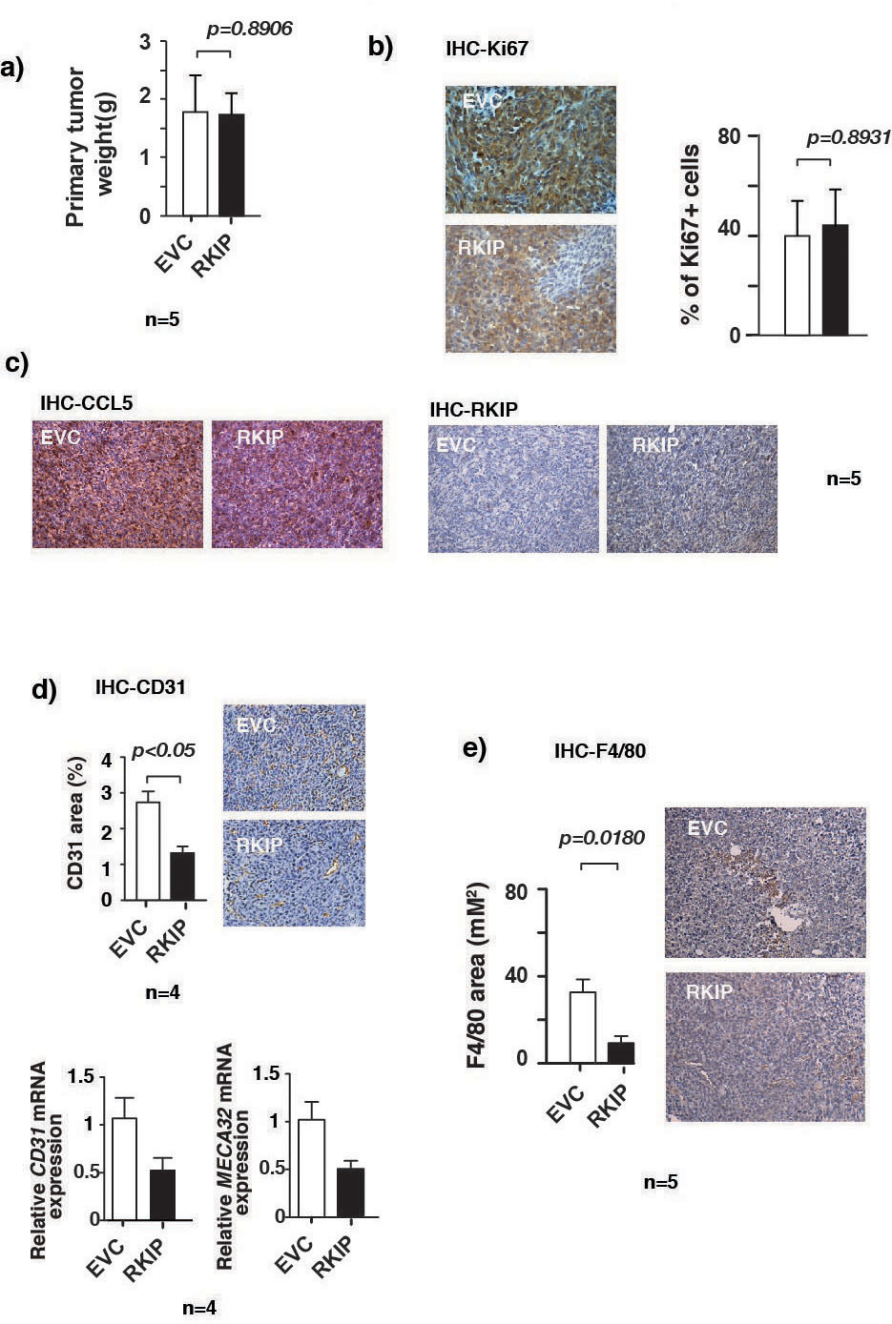
RKIP inhibits angiogenesis and infiltration of macrophages in primary tumors (a) Weights of primary tumors removed from mice injected orthotopically with 4T1 cells stably infected with the indicated retroviral expression vectors. No significant difference in the weights was observed between EVC or RKIP expressing primary tumors as shown by paired t-test, p=0.8906. (b) Immunohistochemical staining for Ki-67 expression in primary tumors with specific Abs. Tumors were excised from mice injected orthotopically with 4T1 cells stably infected with the indicated retroviral expression vectors and processed for staining as described in Materials and Methods. (c) Tumors that were described in (b) were immunohistochemically stained with specific Abs for CCL5 and RKIP. (d) Upper right panel, tumors that were described in c) were also immunohistochemically stained for blood vessels with Ab specific endothelial cell marker CD31. Upper left panel shows quantitation of areas stained positive in the upper right panel for CD31 and reduced staining in RKIP expressing tumors by paired t-test. Lower panel shows the relative *CD31* and *MECA32* mRNA levels assessed by qRT-PCR in control tumors or tumors expressing RKIP proteins. *CyclophilinA* mRNA level was used as internal control. (e) Right panel, immunohistochemical staining for F4/80 macrophage marker in primary tumors shows significantly reduced F4/80 staining in RKIP expressing tumors as compared to control vector expressing tumors as assessed by paired t-test (p=0.0180). Left panel shows quantitation of areas stained positive in the right panel for F4/80.

Metastasis is a complex multimodal activity that involves both cancer cells and surrounding non-transformed cells. It has been shown that the recruitment of non-malignant cells into the tumor microenvironment is essential for tumor growth, and cancer metastases [3]. Expression of RKIP is low in cancer metastases and re-expression of RKIP inhibits cancer metastasis [39]. Besides constraining cancer cell invasion, it is possible that RKIP may also inhibit cancer progression and metastasis by interfering with the communication between cancer and non-transformed cells. To investigate this possibility we used orthotopic breast cancer cell transplantation mouse model to assess the impact of restoring RKIP expression on the number of infiltrating endothelial cells and macrophages in primary tumors. We injected RKIP expressing or control vector 4T1 murine breast cancer cells into the mammary fat pads of 7-week-old female Balb/c mice. At 4 weeks after implantation primary tumors were harvested for analyses. We detected the presence of vascular endothelial cells and macrophages in the primary tumors by IHC staining with CD31 and F4/80 antibodies, respectively. Consistent with its tumor suppressive function, RKIP expression reduced significantly the number of infiltrated macrophages and CD31 positive endothelial cells in the primary tumors as detected by IHC staining (Fig. 4c, upper panel and 4d). As a complementary approach, we also quantified the expression of MECA32 (another endothelial marker) and CD31 transcripts in primary tumors by qRT-PCR (Fig. 4c, lower panel). As previously reported [23], at the time of harvest we observed no statistically significant difference in the weight of the primary tumors of mice injected with control and those of mice injected with RKIP 4T1 cells (Fig. 4a). Concurring with a decrease in CCL5 mRNA expression in RKIP expressing breast cancer cells (Fig. 1a), we also observed a decrease in CCL5 protein expression in RKIP-expressing 4T1 tumor (Fig. 4b) by immunohistochemical (IHC) staining with CCL5 specific Ab.

**Figure 5:**
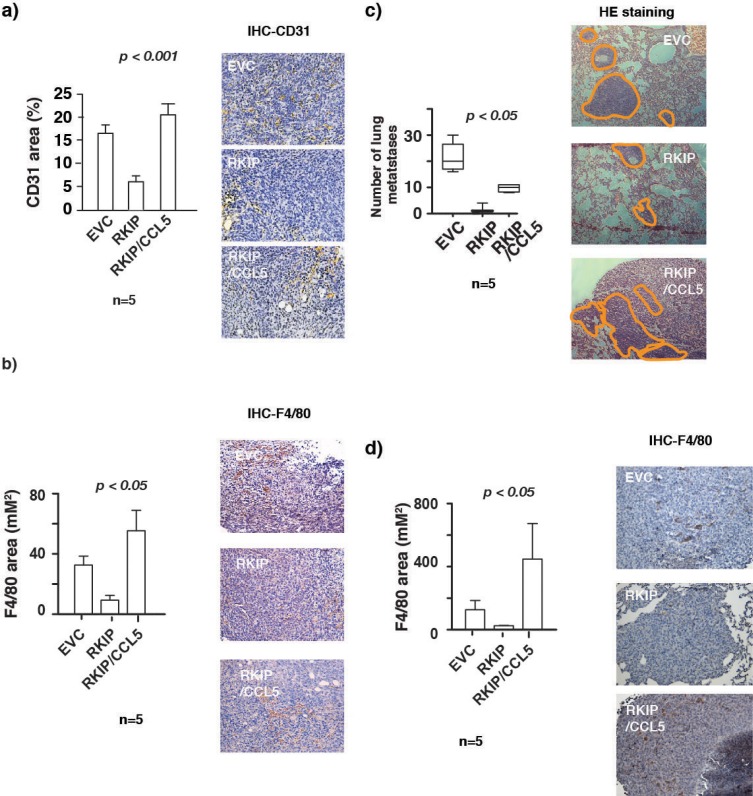
RKIP inhibits lung metastasis and infiltration of TAMs in primary tumors and lung metastatic nodules by inhibiting CCL5 expression (a) Right panel, immunohistochemical staining for CD31 in control or primary tumors expressing RKIP or RKIP and CCL5 together. Left panel shows quantitation of areas stained positive in the right panel for CD31. (b) Right panel, immunohistochemical staining for F4/80 in control or primary tumors expressing RKIP or RKIP and CCL5 together. Left panel shows quantitation of areas stained positive in the right panel for F4/80. One way analysis of variance was performed with a P-value=0.0186. It was followed by Newman-Keuls multiple comparison test where RKIP vs RKIP/CCL5 were found to be significant with a p-value<0.05. (c) Left panel shows number of metastatic nodules in lungs removed from mice injected orthotopically with 4T1 cells stably infected with the indicated retroviral expression vectors. One way analysis of variance was performed with a P-value<0.0001. It was followed by Newman-Keuls multiple comparison test EVC vs RKIP and RKIP vs RKIP/CCL5 were found to be significant. Right panel, H&E stained sections of lungs removed from mice bearing tumors control or tumors expressing RKIP or RKIP and CCL5 together. The macro-metastatic nodules were outlined with orange lines. (d) Right panel, immunohistochemical staining for F4/80 in control or lung metastatic nodules expressing RKIP or RKIP and CCL5 together. Left panel shows quantitation of areas stained positive in the right panel for F4/80. Kruskal-Wallis analysis was performed with a P-value=0.0038. It was followed by Dunn's multiple comparison test where EVC vs RKIP and RKIP vs RKIP/CCL5 were found to be significant with a p-value<0.05.

CCL5 is known to recruit macrophages at the site of the tumor. Once recruited, macrophages can in turn secrete angiogenetic factors essential for construction of new vasculature fueling further tumor growth and the subsequent metastasis. Here, we show that RKIP regulates the expression of CCL5. Our results therefore prompt us to investigate whether the shutting down of CCL5 expression by RKIP is the cause of the observed reduction in the infiltration of macrophages and vascular endothelial cells into the primary tumors. Indeed, we observed that expression of CCL5 was able to rescue the effect of inhibition of macrophage recruitment and decrease of CD31 positive cells in RKIP-expressing primary tumors (Fig. 5a-5b). Thus, our results indicate that RKIP inhibits infiltration of macrophages and endothelial cells in the primary tumors mechanistically by suppressing the expression of CCL5.

### RKIP inhibits lung metastasis and lung macrophage infiltration by inhibiting CCL5 expression

RKIP is a known metastasis suppressor and we observed a decrease in lung metastasis in mice injected with RKIP expressing 4T1 cells (Fig. 5c) as has been previously reported in murine breast cancer orthotopic models [21, 23, 24]. However, the mechanism of how RKIP blocks metastasis of breast cancer cells to the lung has not been rigorously investigated. Lung infiltration with monocytic cells including macrophages has been found to promote breast cancer cells lung tropism in experimental mouse models [40, 41]. Our results therefore raise the possibility that RKIP inhibits metastasis by reducing the macrophages lung infiltration through negative regulation of CCL5 expression. Truly, while expression of RKIP inhibited lung metastasis and macrophage infiltration, co-expression with CCL5 was able to restore macrophages lung infiltration and partially rescued the inhibition of lung colonization mediated by RKIP (Fig. 5c-5d). These results indicate that RKIP suppresses lung colonization by inhibiting the expression of CCL5 thereby blocking macrophage infiltration.

## DISCUSSION

Although stepwise accumulation of mutations in oncogenes and tumor suppressor genes in cancer cells remains the driving force for tumor initiation and progression, tumors require the support of surrounding non-transformed cells to survive, proliferate and progress. This heterotypic interaction of malignant cells with non-malignant cells is essential for tumors to grow, resist cell death, induce angiogenesis, invade tissues and form metastases. The continual interaction and active recruitment of non-malignant cells from adjacent stromal tissues are required for a sustained supportive-system for cancer development [2, 3]. Non-malignant cells found in the milieu in which a tumor develops can be of many types including infiltrating immune cells, cancer-associated fibroblastic cells and angiogenetic vascular cells [3]. Among the immune cells, macrophages are highly represented. Furthermore, there is mounting clinical and experimental evidence that these tumor-infiltrating macrophages play a pivotal role in enhancing tumor progression and subsequent metastasis to distant sites [5]. It was shown previously that RKIP is a significant suppressor of cancer metastasis. Expression of RKIP is low in cancer metastasis. Although primary tumor growth was unaffected, re-expression of RKIP inhibits cancer metastasis [39]. The functional mechanism of how RKIP inhibits metastasis remains undefined. In this study, we observed an inverse correlation of the number of TAM and RKIP expression in both primary tumors and lung metastases in an immunocompetent orthotopic breast cancer mouse model. Our present study therefore suggests that RKIP may inhibit metastasis by interfering with capacity of cancer cells to recruit macrophages.

Once recruited into the primary tumor macrophages secrete growth factors and proteases to facilitate tumor cells invasion. Equally as important is the production of vascular endothelial growth factor and other angiogenetic molecule to stimulate tumor-associated neovasculature growth (angiogenesis) [42]. Previously it was shown that in an orthotopic prostate cancer mouse model that expression of RKIP inhibited tumor angiogenesis [13]. However, the molecular mechanism has not been thoroughly examined. Here, we show that expression of RKIP decreases the number of CD31 positive endothelial cells in breast cancer allografts. Our results therefore suggest, for the first time, the angiogenesis suppressive function of RKIP in breast cancer. Since macrophages are critical for angiogenesis, it is possible that one of the mechanisms by which RKIP inhibits angiogenesis could be by indirectly hindering macrophages infiltration into the tumor.

Cancer is a systemic disease. Primary tumors can impact metastatic outcome by secreting factors that cause accumulation of myeloid cells like macrophages at distant sites [43]. It has been shown experimentally that macrophages are important for setting up preferred sites for metastatic seeding as well as enhancing tumor cells extravasation and subsequently successful colonization at distant site. Here, we show that expression of RKIP decreases both the number of lung metastases and the number of macrophages present in the metastases. Our results therefore suggest that RKIP may inhibit lung metastasis and colonization by limiting the access of macrophages to the lungs.

Cancer cells aberrantly secrete a whole battery of growth factors and chemokines such as CCL2 and CCL5 to recruit macrophages to the tumor microenvironment where they are stimulated to mature and secrete further bioactive molecules to aid processes crucial for cancer development and metastasis [9, 10]. We observed that in established human and mouse breast cancer cell lines the expression level of CCL5 was negatively regulated by RKIP. While knocking down of RKIP expression increased CCL5 expression, restoration of RKIP expression in low-RKIP-expressing breast cancer cells decreased CCL5 expression. This causal negative role of RKIP on CCL5 expression is clinically relevant because a statistically significant negative correlation of RKIP and CCL5 expression was also observed in breast cancer clinical samples. We have previously showed that RKIP regulated expression of multiple chemokines in lung cancer cell line A549 through the IKK-NF-kB transcriptional axis [37]. It is therefore possible that RKIP regulates the expression of CCL5 by a similar mechanism in breast cancer cells.

Taken together, it is possible that RKIP inhibits breast cancer metastasis by interfering with the macrophage recruitment by shutting down CCL5 expression by the cancer cells. Indeed, in our 4T1 breast cancer mouse model, ectopic expression of CCL5 was sufficient to reverse the inhibition of angiogenesis and macrophage recruitment due to RKIP expression. Lastly, CCL5 expression also partially rescued the RKIP-mediated inhibition of lung metastasis. This partial-rescue phenotype suggests that RKIP may inhibit cancer metastasis by impinging on multiple targets.

In addition to functioning as macrophage chemotactic factor, the secreted CCL5 can also act in an autocrine manner to enhance the invasive capacity of cancer cells [8, 38]. Previously, we showed that RKIP expression negatively correlated with the invasive capacity of the breast cancer cells *in vitro* [23]. While restoration of RKIP in low-RKIP-expressing invasive cancer cells suppressed CCL5 expression and inhibited invasion, silencing of RKIP in high-RKIP-expressing non-invasive cells increased CCL5 expression. Since CCL5 stimulates invasion of breast cancer cells, it is possible that RKIP inhibits cancer cells invasion by decreasing CCL5 expression and silencing of RKIP increases invasion because of the increased CCL5 expression. Our loss- and gain-of-function CCL5 expression rescue experiments indeed demonstrated the causal role of CCL5 in RKIP-mediated regulation of breast cancer cells invasion *in vitro*. The *in vivo* metastatic process is a complex cascade that consists of distinct steps. RKIP expression inhibits several earlier steps of the cascade including local invasion, angiogenesis, intravasation as well as extravasation and colonization [21, 24]. Here, we show that RKIP inhibits angiogenesis and colonization by targeting CCL5. It remains to be determined whether ectopic expression of CCL5 can also reverse the RKIP-mediated inhibition of local invasion *in vivo*.

To summarize, our work has attributed yet another function to RKIP as a metastasis suppressor by regulating the expression of chemokine CCL5. Through the inhibition of CCL5 expression, our study has for the first time discovered the causal role of RKIP in modulating tumor microenvironment by disrupting the communication between cancer cells and macrophages. Since the tumor-macrophage crosstalk potentiates cancer invasion and metastasis, this newly identified pathway involving RKIP may provide new druggable targets for therapeutic intervention.

## MATERIAL AND METHODS

### Cell line authentication

MDA-MB231 subline 4175 was validated by measuring the expression of the subset of genes from the lung metastasis gene signature associated with these cells as previously reported [26].

4T1 and 4T07 cells were authenticated for their resistance to thioguanine and diaminopurine, respectively [27]. In addition upon orthotopic transplantation into Balb/c mice, 4T1 spontaneously metastasizes to the lung, whereas 4T07 are highly tumorigenic but fail to metastasize at different points in dissemination [28]. MCF7, MCF10A and BT20 cells were validated by measuring the expression of EMT associated genes as previously reported [29].

### *In vitro* invasion assay

The polycarbonate membrane (8μΜ pore size) of FluoroBlok cell culture inserts (BD Biosciences) was coated with 95 μL (for MDA-MB-231_4175) or 50 μL(for BT20) of Matrigel (1:26) (BD Biosciences) and was incubated at 37°C. MDA-MB-231_4175 (serum starved for 4 hours) or BT20 cells (serum starved overnight) were plated at 4.5×104 or 5×103 seeding densities respectively on matrigel coated inserts. 700μL of chemo-attractive medium (Dulbecco's Modified Eagle's medium, 1% P/S and 10% FBS) was added to the lower chambers (24-well BD Falcon TC companion plate). After 24 or 48 hours of incubation, the insert bottoms were dipped in 1X PBS and stained in Calcein AM reconstituted in DMSO to 1mg/mL- 1μl in 700 μl 1X PBS (BD Biosciences). Digital images were captured on EVOS inverted microscope along with manual cell count and fluorescence reading (485-538nM). Invasion assays were performed at least twice in triplicates. One way ANOVA was performed followed by Bonferron's multiple comparison test.

### Mouse mammary fat pad injection and post-injection harvesting of tissues

All animal work was performed in accordance with an IACUC (Institutional Animal Care and Use Committee) approved protocol. Mammary fat pad injection was performed as described previously [30]. Briefly, female BALB/cJ mice (7 weeks old) were purchased from Jackson Laboratory. The animals were anesthetized using Ketamine-Xylazine cocktail (10:1 ratio). Incision was made so as to expose mammary fat pad number 9. 1×105 control/RKIP overexpressing / RKIP-CCL5 overexpressing/overexpressing 4T1 cells in 10 μl phosphate-buffered saline, pH 7.4, were injected into the mammary fat pad. The incision was closed with wound-clips (Harvard Apparatus). A week later the wound clips were removed. Following 4 weeks post-injection, tissues were harvested for cancer metastasis analyses.

#### Primary tumor harvesting

Primary tumors were dissected out, weighed and preserved for RNA extraction and histological analyses.

#### Quantification of lung metastasis

Lungs were dissected out after euthanasia and macro-metastatic nodules were counted. Lungs were paraffin-embedded for histochemical analysis. One way analysis of variance was performed followed by Newman-Keuls multiple comparison test.

### Immunohistochemistry

Formalin fixed paraffin embedded tumors and lungs were cut into 5μ and stained for CD-31 an angiogenesis marker, CCL5, RKIP, Ki-67 and F4/80 macrophage marker. Briefly, the sections were deparaffinized followed by heat-mediated retrieval at 85°C in a water bath for 45 mins in citrate buffer pH-6.0. The sections were allowed to sit at RT for at least 30 mins. This was followed by blocking of endogenous peroxidase activity in H202-methanol. The sections were then blocked using normal goat serum (Vector labs ABC Kit) followed by incubation in primary antibody at 4°C overnight. Next day, after washing of primary antibody, sections were incubated in anti-mouse biotinylated secondary antibody (Dako) for 30 mins at RT. This was followed by incubation with Avidin-Biotin (VectaStain ABC Kit) complex and chromogenic detection using the DAB-peroxidase kit (VectaStain peroxidase Kit). Brightfield images were taken at 20X and 40X magnification.

The area of F4/80 positive staining in primary tumors was measured using NIS elements software. Percentage areas were then calculated. One way analysis of variance was performed followed by Newman-Keuls multiple comparison test. Quantification of F4/80 staining in lungs was restricted only to lung metastatic nodules. Two to three fields of most lung nodules in an entire section were quantified. For statistical analysis, Kruskal-Wallis test was performed followed by Dunn's multiple comparison test. For Ki-67 staining quantification, the number of positively stained nuclei and the total number of nuclei per field (total of two random fields per sample) were counted at 40X magnification. Percentage positive nuclei were counted regardless of the staining intensity. Student's t-test was performed and no significant difference was observed between EVC and RKIP expressing primary tumors.

### Data preprocessing of Affymetrix microarray gene expression

Data preprocessing of Affymetrix microarray gene expression was described previously [31]. Briefly, 26 cohorts constituting 3,992 microarray gene expression of human breast cancer on Affymetrix U133A or U133Plus2 platforms were downloaded from Array Express, Gene Expression Omnibus (GEO) and author's website [32]. Robust Multichip Average (RMA) normalization was performed on each dataset. The normalized data was combined and subsequently standardized using ComBat [33] to remove batch effect. Breast cancer subtypes were inferred based on enrichment score of a subtype signature [34, 35] computed by Single sample Gene Set Enrichment Analysis (ssGSEA) [35], as described previously [31].

Similarly, GSE15026 (n=30 samples corresponding to 19 cell lines) and E-TABM-157 (n=51 samples corresponding to 51 cell lines) were downloaded from GEO and were subjected to the same pre-processing as the clinical samples. The RMA-normalized and ComBat-standardized breast cancer cell line panel consists of 81 samples corresponding to 70 cell lines. Subtype of breast cancer cell lines was taken from [36] based on the cell line name. Basal-B subtype in the original paper [36] was labeled as Claudin-low in this study.

Epithelial-mesenchymal transition (EMT) score for breast cancer tumor and cell line were computed based on method and signature derived previously [29].

### Statistical analysis

Statistical significance evaluation by Mann-Whitney test and Spearman correlation coefficient test were computed using Matlab® version 7.14.0.739 (R2012a).

## SUPPLEMENTARY MATERIAL METHODS AND FIGURE


