# Correction: RKIP regulates CCL5 expression to inhibit breast cancer invasion and metastasis by controlling macrophage infiltration

**DOI:** 10.18632/oncotarget.9139

**Published:** 2016-02-05

**Authors:** Ila Datar, Xiaoliang Qiu, Hong Zhi Ma, Miranda Yeung, Shweta Aras, Ivana de la Serna, Fahd Al-Mulla, Jean Paul Thiery, Robert Trumbly, Fan Xuan, Hongjuan Cui, Kam C. Yeung

Present: Due to an error made by the authors while submitting a revision, Dr. Tuan Zea Tan was omitted from the list of authors.

Corrected: Correct author list can be found below. Authors sincerely apologize for this oversight.

Original article: Oncotarget. 2015; 6(36): 39050-61. doi: 10.18632/oncotarget.5176.

**Ila Datar^1^, Xiaoliang Qiu^1^, Hong Zhi Ma^1^, Miranda Yeung^1^, Shweta Aras^1^, Ivana de la Serna^1^, Fahd Al-Mulla^2^, Tuan Zea Tan^3^, Jean Paul Thiery^3,4^, Robert Trumbly^1^, Fan Xuan^5^, Hongjuan Cui^5^ and Kam C. Yeung^1^**

^1^ Department of Biochemistry and Cancer Biology, University of Toledo, College of Medicine, Health Science Campus, Toledo, OH, USA

^2^ Kuwait University, Faculty of Medicine, Safat, Kuwait

^3^ Cancer Science Institute of Singapore National University of Singapore, Center for Translational Medicine, Singapore, Singapore

^4^ Department of Biochemistry, Yong Loo Lin School of Medicine, National University of Singapore, Singapore, Singapore

^5^ State Key Laboratory Of Silkworm Genome Biology, Chongqing, China

