# Supplementary material for: RKIP regulates CCL5 expression to inhibit breast cancer invasion and metastasis by controlling macrophage infiltration
Source: Oncotarget. 2015 Aug 13;6(36):39050–61. doi: 10.18632/oncotarget.5176 (PMC4770756; doi:10.18632/oncotarget.5176)
Supplement: Supplementary file 1 [file oncotarget-06-39050-s001.pdf]

RKIP regulates CCL5 expression to inhibit breast cancer invasion and metastasis By controlling macrophage infiltration

Supplementary Material

Extended Materials and Methods

Cell lines and reagents:

Matrigel was from BD Biosciences. The highly metastatic lung-tropic MDA-MB231 subline 4175 was kindly provided by Dr. J. Massague [1]. MCF10A, 4T1, and 4T07 cells were kindly provided by Dr. Fred Miller (Karmanos Cancer Institute, MI). BT20 and MCF7 were purchased from ATCC. 4T1, and 4T07 cells were cultured in Dulbecco's modified Eagle's medium with 5% FBS, 5% newborn calf serum and 1% pen-strep. BT20, MCF7, and MDA-MB-231_4175 cells were cultured in Dulbecco's modified Eagle's medium with 10% FBS. Cells were grown in a humidified tissue culture incubator at 37°C in 5% CO_2.

The human CCL5 siRNA lentiviral expression constructs were purchased from GeneCopoeia. Rabbit anti-RKIP antibody was from Invitrogen, and mouse monoclonal anti-α-tubulin antibody was from Sigma. CCL5 antibody was from Acris. Horseradish peroxidase secondary antibodies were from Santa Cruz Biotechnology. F4/80 anti-rat antibody was from Pierce (Thermo scientific). CD-31 antibody was from Abcam. Biotinylated secondary anti-mouse antibody was from Dako. Biotinylated secondary anti-rabbit and anti-rat antibodies were from Vector labs.

Preparation of knockdown cells:

For RNAi-based silencing of expression of RKIP in 4T07 and BT20 cell lines, we prepared knockdown cells expressing the corresponding small interfering RNAs (siRNAs) or a firefly luciferase-specific siRNA as control as previously described [2-4]. Two siRNAs targeting two different regions of the RKIP

sequence were used. Knockdown was confirmed by Western blot analysis or qRT-PCR (Supplementary Figure, panel a). Knockdown of RKIP expression in 4T07 or BT20 cells by either siRNA had similar effects on CCL5 expression and cancer cell invasion *in vitro* (Fig. 1b and Supplementary Fig.). One of the two constructs was used in our study.

Preparation of RKIP and CCL5 expressing cells:

Different cDNAs were ectopically expressed individually or in combination in 4T1 or MDA-MB-231 cells by retroviral-mediated gene transfer as previously described [2]. cDNA expression was confirmed by Western blot analysis or qRT-PCR.

Cell extracts and Western Blot analysis:

Cells extracts were prepared and Western blotting was carried out as previously described [4]. Briefly, cells were lysed with 20 mMTris, pH 7.4, 150mM NaCl, 2mM EDTA, and 1% Triton X-100. Samples (10–50 μg protein) were separated by sodium dodecyl sulfate-polyacrylamide electrophoresis and then electrophoretically transferred from the gel to polyvinylidene fluoride membranes (Millipore). The primary antibodies (anti-RKIP or anti-α -tubulin) was diluted in phosphate buffered saline, pH 7.4, 0.2% Tween-20, 5% bovine serum albumin, 0.002% sodium azide. Following three washes, blots were incubated with the appropriate horseradish peroxidase-conjugated secondary antibody for 1 h at room temperature. Proteins were visualized with enhanced chemilluminescence with the BioRad ChemiDoc EQ system.

Quantitative real-time reverse transcriptase-polymerase chain reaction (qRT-PCR):

Total cellular RNA was extracted with QIAzol reagent (Qiagen) and reverse transcribed with random hexamer primers (Applied Biosystems). The resulting cDNAs were used for PCR with the SYBR-Green Master PCR mix (Qiagen) in triplicate. PCR and data collection were performed on an ABI 7500 Real time thermal cycler (Applied Biosystems). β-actin or glyceraldehyde-3-phosphate dehydrogenase was used as an internal control.

zRKIP-F	GGGGTGACGGTGGACGAGCT
zRKIP-R	CCTGCTGGGAGCATCGGGGT
hCXCL2-F	AGTGGCAAATCCAACTGACC
hCXCL2-R	AAACACATTAGGCGCAATCC
hCCL5-F	CCAGCAGTCGTCTTTGTCAC
hCCL5-R	CTCTGGGTTGGCACACACTT
mCCL5-F	GCTGCTTTGCCTACCTCTCC
mCCL5-R	TCGAGTGACAAACACGACTGC
mCD31-F	CTGCCAGTCCGAAAATGGAAC
mCD31-R	CTTCATCCACCGGGGCTATC
h□-ACTIN-F	CACTGTGTTGGCGTACAGGT
h□-ACTIN-R	TCATCACCATTGGCAATGAG
hGAPDH-F	TGCACCACCAACTGCTTAGC
hGAPDH-R	GGCATGGACTGTGGTCATGAG
m□-ACTIN-F	ATCTGGCACCAGACCTTCTACAATGAGCTGCG
m□-ACTIN-R	CGTCATACTCCTGCTTGCTGATCCACATCTGC
mGAPDH-F	TTCACCACCATGGAGAAGGC
mGAPDH-R	GGCATGGACCGTGGTCATGA

CCL5 ELISA Assay:

$2x10^6$ cells were seeded in 6cm cell culture dish with 3ml of DMEM media supplemented with 10% FBS. The cells were incubated at 37°C, 5% CO_2 for 24hrs. The old media were removed and replaced with 2ml of fresh media. The cells were incubated for another 20hrs before the media were harvested. Cell culture media were centrifuged at 1500 rpm for 10 minutes to

remove debris. Cell culture supernatants were stored in -80°C for later use. The samples were concentrated before the ELISA assay using Amicon® Ultra-4 centrifugal filters – 3K (MilliPore UFC800324). The samples were centrifuged at 4000xg at room temperature for 35 minutes. 7ml samples were concentrated to 330ul.

The CCL5 protein was detected by using the RANTES (CCL5) Human SimpleStep ELISA ™ Kit from Abcam® according to the manufacturer instructions. 100µl of samples and standards were added to appropriate wells. 100 µl of Antibody Cocktail were added to each well. The 96-well plate reader Molecular Devices Spectramax M5 reads the assay endpoint at 450nm. Data were presented as fold expression ± SEM. Student t-test was performed.

References:

[1] Minn AJ, Gupta GP, Siegel PM, Bos PD, Shu W, Giri DD, et al. Genes that mediate breast cancer metastasis to lung. Nature. 2005;436:518-24.

[2] Beshir AB, Ren G, Magpusao AN, Barone LM, Yeung KC, Fenteany G. Raf kinase inhibitor protein suppresses nuclear factor-kappaB-dependent cancer cell invasion through negative regulation of matrix metalloproteinase expression. Cancer Lett. 2010;299:137-49.

[3] Park S, Yeung ML, Beach S, Shields JM, Yeung KC. RKIP downregulates B-Raf kinase activity in melanoma cancer cells. Oncogene. 2005;24:3535-40.

[4] Tang H, Park S, Sun SC, Trumbly R, Ren G, Tsung E, et al. RKIP inhibits NF-kappaB in cancer cells by regulating upstream signaling components of the IkappaB kinase complex. FEBS Lett. 2010;584:662-8.

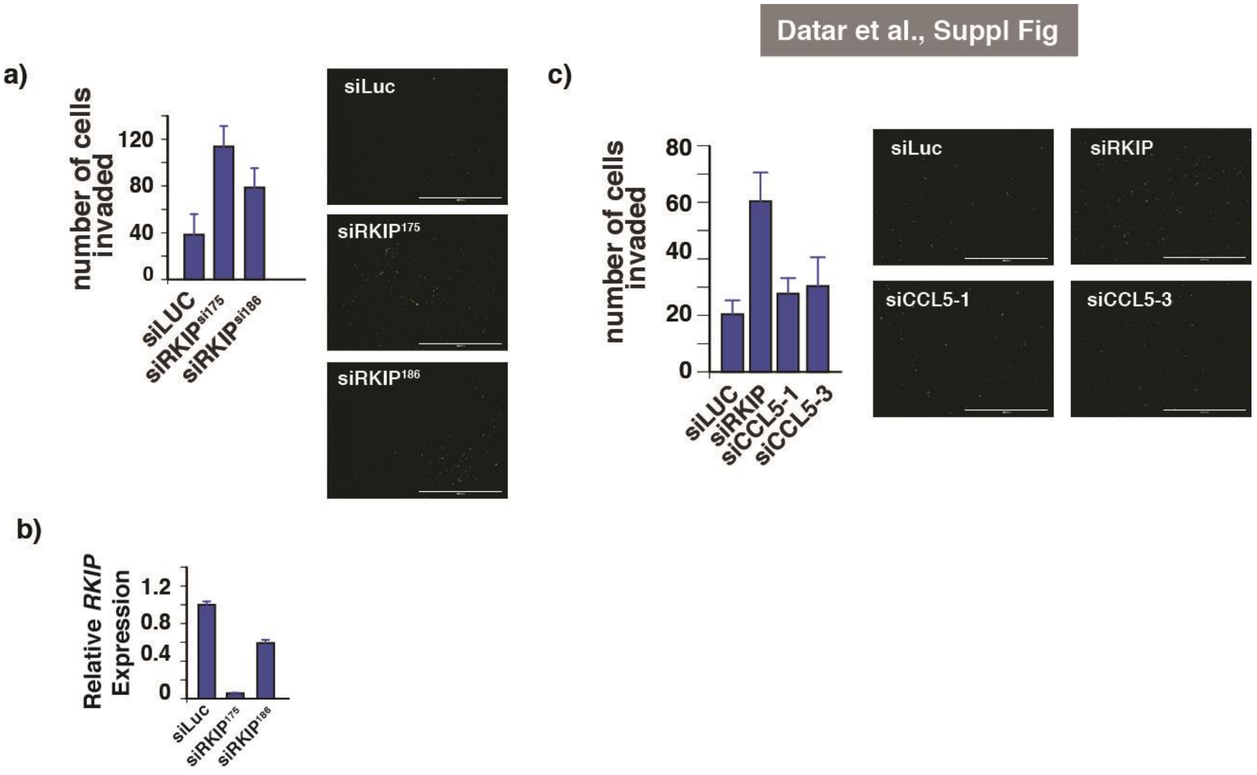

Supplementary Figure

(a) Invasion Assay of BT20 breast cancer cells showing increased invasion upon RKIP knockdown using two different siRNAs. Number of cells invaded through the inserts were stained with green fluorescent stain, calcein and counted 48 hours after plating (Left panel). Right panel shows representative images of the respective matrigel inserts. (b) RKIP mRNA expression upon knockdown using two different siRNAs as detected by qRT-PCR. (c) Invasion assay of BT20 cells showing no significant difference between the invasive capacity of control knockdown versus CCL5 knockdown using two different siRNAs. Number of cells invaded through the inserts were stained and counted as described in (a). Right panel shows representative images of the respective matrigel inserts.
